# Fibroblast Growth Factor 21 in Chronic Hepatitis C: A Potential Non-Invasive Biomarker of Liver Status upon Viral Eradication

**DOI:** 10.3390/metabo13111119

**Published:** 2023-10-30

**Authors:** Filippo Biagi, Francesco Carlomagno, Martina Carbone, Roberta Veralli, Umberto Vespasiani-Gentilucci, Elisabetta Riva, Silvia Manfrini, Dario Tuccinardi, Adriano De Santis, Lucio Gnessi, Mikiko Watanabe

**Affiliations:** 1 Department of Experimental Medicine, Section of Medical Pathophysiology, Food Science and Endocrinology, Sapienza University of Rome, 00161 Rome, Italyfrancesco.carlomagno@uniroma1.it (F.C.); mikiko.watanabe@uniroma1.it (M.W.); 2 Department of Experimental and Clinical Medicine, University of Florence, 50134 Florence, Italy; 3 Department of Translational and Precision Medicine, Sapienza University of Rome, 00185 Rome, Italyadriano.desantis@uniroma1.it (A.D.S.); 4 Department of General Surgery, Section of Gastroenterology, Azienda Sanitaria Universitaria Friuli Centrale–P.O. Santa Maria della Misericordia di Udine, 33100 Udine, Italy; 5 Clinical Laboratory Unit, Fondazione Policlinico Universitario Campus Bio-Medico, 00128 Rome, Italy; r.veralli@policlinicocampus.it; 6 Unit of Virology, Fondazione Policlinico Universitario Campus Bio-Medico, 00128 Rome, Italy; 7 Unit of Clinical Medicine and Hepatology, Area of Medicine, Campus Bio-Medico University of Rome, 00128 Rome, Italy; 8 Department of Medicine and Surgery, Campus Bio-Medico University of Rome, 00128 Rome, Italy; 9 Department of Endocrinology and Diabetes, Campus Bio-Medico University of Rome, 00128 Rome, Italy

**Keywords:** cirrhosis, DAAs, FGF-21, HCV, NAFLD, NASH

## Abstract

Fibroblast growth factor 21 (FGF-21), previously recognized as a marker of liver damage and a potential drug target in non-alcoholic fatty liver disease (NAFLD), has unclear implications in hepatitis C virus (HCV) infections. This study aimed to investigate the relationship between FGF-21 levels and liver health in patients with HCV undergoing direct-acting antiviral (DAA) treatment. Forty-five patients were assessed for liver stiffness, blood chemistry, and other relevant metrics before and after achieving sustained viral response (SVR), defined as the absence of detectable HCV-RNA after 24 weeks of treatment. Post-treatment, all patients showed a decrease in liver stiffness and improved liver enzyme levels (AST and ALT), alongside an increase in FGF-21 levels. Interestingly, the increase in FGF-21 correlated negatively with liver stiffness but showed no correlation with hepatic steatosis. The observed elevation in FGF-21 levels at SVR following DAA therapy for chronic HCV infection can be attributed to the restoration of hepatic function, including its synthetic capabilities. Specifically, the mitigation of liver fibrosis post-HCV eradication is expected to lead to improvements in liver function, such as enhanced albumin and FGF-21 production. This improvement in synthetic function likely drives the increase in FGF-21 levels, rather than changes in liver fat content. We suggest a potential role of FGF-21 as a marker of fibrosis and hepatic cytotoxicity and as a drug target beyond NAFLD, to be confirmed by additional studies.

## 1. Introduction

Hepatitis C virus (HCV) is still one of the major causes of chronic liver disease and, although in recent years non-alcoholic steatohepatitis (NASH) has become the leading cause of liver disease and indication for liver transplant in women, HCV-related hepatopathy remains an important burden worldwide [1,2,3,4]. Although most of the attention has been placed on liver-related complications [5,6], HCV-induced metabolic derangement determines an important contribution to morbidity and mortality in the setting of chronic hepatitis C (CHC), even after its eradication [7,8]. Indeed, the persistence of hepatitis C virus has been linked to the pathogenesis of type 2 diabetes mellitus, atherosclerosis, and steatosis, all of which are a frequent finding in chronic HCV infection [9]. Although hepatic steatosis is a very common histological finding, regardless of the genotype, a particularly strong association has been reported with infection by genotype 3, one of the seven major known HCV genotypes, during which mean steatosis tends to be higher with respect to other genotypes [9,10,11]. Furthermore, this connection is confirmed by the reduction of adipocyte accumulation after eradication treatment and worsening of steatosis with infectious relapses [9,12].

Among the several players and pathways involved in lipid dysmetabolism in CHC, one of the key mechanisms that appears to induce hepatic steatosis development is the inhibition of peroxisomal proliferator activated receptor α (PPAR-α) [13,14]. Several studies have described the correlation between PPAR-α and HCV infection, reporting a decreased expression of said receptor [15,16]. Additional evidence from an in vitro study conducted on liver specimens from genotype 1 HCV-infected patients, in addition to confirming the underexpression of PPAR-α, described a negative correlation between HCV-RNA and fibroblast growth factor 21 (FGF-21) mRNA levels, with FGF-21 being a hepatokine primarily produced by the liver, and one of PPAR-α’s target genes [13]. Several studies reported the ability of PPAR-α agonists to reduce HCV-RNA levels [17], and with FGF-21 being one of PPAR-α’s target genes, this pathway could be exploited for therapeutical purposes. Given the elevated prevalence of metabolic syndrome and its hepatic manifestation, in the general population, differentiating between hepatic steatosis due to viral infection (viral steatosis) and hepatic steatosis due to a coexisting metabolic syndrome (metabolic steatosis) remains a challenge [9]; however, with the PPAR-α–FGF-21 axis being involved in the pathogenesis of both, the administration of FGF-21 analogues could be of benefit without the need to discriminate between the two.

With the introduction of direct-acting antiviral (DAA)-based treatment protocols, viral eradication has become possible, reducing the risk of long-term complications associated with a chronic infection [18]. Although these recent advances have drastically improved the prognosis of HCV infection, eradication through mass vaccination remains an open challenge due to the impressive genetic heterogeneity of the virus [19,20]. The structural and functional alterations caused by the chronicization of the infection, mostly mediated by immune-mediated processes rather than the cytopathic effects of the virus itself, result in extracellular matrix (ECM) deposition and fibrosis, which provides an estimate of the risk of progression [21,22]. To date, the gold standard for fibrosis and NAFLD detection and staging is liver biopsy, a solid yet invasive approach associated with some complications. Due to its limitations such as sampling error, invasiveness, interobserver variability among pathologists, and the fact that it provides a static evaluation, non-invasive approaches have been largely investigated. In particular, a large number of non-invasive methods to assess fibrosis, both direct and indirect, have been proposed in the setting of HCV infection [23]. Among them, FGF-21 has already been proposed as a biomarker in NAFLD, with predictive relevance of hepatic damage as a result of steatosis [24,25,26]. Compared to healthy subjects, FGF-21 appears to be higher in cirrhotic patients and various hepatic insults with some studies reporting a positive correlation between FGF-21 and the degree of necro-inflammation and fibrosis [25,27,28,29,30]. With FGF-21 being a relatively well-established marker of liver damage, it is not necessarily surprising that a previous study investigating patients with chronic HCV infection reported a decrease in FGF-21 12 weeks after DAA viral eradication [31]. However, this finding was in contrast with the previous in vitro studies showing a negative correlation between liver HCV and FGF-21 expression [13]. The PPAR-α–FGF-21 axis, as a potential collateral target of HCV treatment, warrants further investigation, and we thus aimed to evaluate circulating FGF-21 in patients with chronic HCV infection and to clarify its trend upon viral eradication with DAAs and the improvement of hepatic health.

## 2. Materials and Methods

### 2.1. Study Design and Treatment Protocol

This was a multicenter, observational, prospective, longitudinal study. Each patient included in the study was monitored for 9 to 12 months, depending on the treatment protocol adopted. Patients were evaluated before starting eradication treatment for HCV, were then treated with a course of second-generation DAAs for a total duration of 12 or 24 weeks and re-evaluated 24 weeks after reaching sustained viral response (SVR, defined as undetectable HCV-RNA), ultimately 9 or 12 months after the first evaluation depending on the antiviral regimen undertaken. According to guideline recommendations and based on the extent of liver disease, the DAAs administered were either the combination sofosbuvir/velpatasvir 400 mg/100 mg (one tablet once daily), elbasvir/grazoprevir 50 mg/100 mg (one tablet once daily), or glecaprevir/pibrentasvir 100 mg/40 mg (three tablets once daily) [32,33]. Eligibility for treatment was assessed according to current clinical practices, assessing the general health conditions, potential drug interactions, and the patient’s willingness to undergo treatment. Patient information was collected through a structured interview, including marital status, educational qualifications, ethnicity, professional occupation, comorbidities, pharmacological history, liver disease duration, and information on any previous antiviral therapy.

Assessment was made before starting eradication treatment with second-generation direct antivirals and 24 weeks after reaching sustained virological response (SVR24); this happened either 9 or 12 months after the first evaluation, depending on the antiviral regimen undertaken. The definition of eradication of chronic HCV hepatitis was based on the achievement of SVR, consisting of the finding of serum HCV-RNA negativity 24 weeks after the end of antiviral therapy.

### 2.2. Study Population

Subjects were enrolled from among those accessing the hepatology units of Azienda Ospedaliero-Universitaria Policlinico Umberto I and Policlinico Universitario Campus Bio-Medico in Rome. The inclusion criteria were as follows: age ≥ 18 and <75 years old, active HCV infection (HCV-RNA detectable in serum), duration of illness greater than 6 months, clinical indication for antiviral therapy with direct antiviral agents, as per international guidelines, and willingness to undergo treatment. The exclusion criteria were identified in order to avoid potential confounding factors, known to affect FGF-21 secretion: presence of active cancer (liver- or non-liver-related), organ failure (described as NYHA classes 3–4 for heart failure, severe chronic kidney disease eGFR < 30 or dialysis, GOLD classes 3–4 for COPD), other causes of liver damage (e.g., hepatitis B infection or other hepatotropic viruses, autoimmune hepatitis, primary biliary cholangitis, hemochromatosis, excessive alcohol consumption or alcohol abuse disorder, non-alcoholic steatohepatitis, or Wilson’s disease), or history of organ transplant [28,34,35,36,37,38].

The study was approved by the local IRB (prot. CE6659) and conducted in accordance with the Declaration of Helsinki and Good Clinical Practice. Written informed consent was obtained from all study participants before enrollment.

### 2.3. Measurement of Data

All patients were assessed at T0, before starting eradication treatment with second-generation direct antivirals, and at T1, 24 weeks after reaching sustained virological response (SVR24). For some patients, SVR24 occurred at 9 months from T0, whereas for others it occurred at 12 months from T0. The difference in SVR24 timings was due to the difference in treatment duration, depending on the antiviral regimen undertaken. The definition of eradication of chronic HCV hepatitis was based on the achievement of SVR, consisting of the finding of serum HCV-RNA negativity 24 weeks after the end of antiviral therapy.

### 2.4. Biochemical Assessment

Routine biochemical tests were handled according to standard operating procedures. Venous blood samples were collected between 8 and 9 a.m. by venipuncture in fasting patients. Samples were then transferred to the local laboratory and handled according to the local standards of practice. The following assays were measured: white blood cells (WBCs), red blood cells (RBCs), hemoglobin (Hb), platelets (PLTs), aspartate aminotransferase/glutamic oxaloacetic transaminase (AST/GOT), alanine transaminase/glutamic pyruvic transaminase (ALT/GPT), total bilirubin, international normalized ratio (INR), albumin, and creatinine. The hepatic steatosis index (HSI) was calculated according to Lee et al.: HSI = 8 × (ALT/AST ratio) + body mass index (BMI + 2 (if female) + 2 (if diabetes mellitus) [39]. FGF-21 was measured using a commercially available assay according to the manufacturer’s protocol (Quantikine human FGF-21, R&D Systems, Inc. Minneapolis, MN, USA).

Quantitative HCV-RNA levels were assessed at baseline, before starting DAAs, and again at the end of treatment (12 or 24 weeks depending on treatment protocol). HCV-RNA was detected using COBAS AmpliPrep/COBAS TaqMan HCV Test, v2.0 (Roche Diagnostics, Alameda, CA, USA). Genotyping was performed using the real-time HCV genotype II assay (Abbott Molecular, Des Plaines, IL, USA), according to the manufacturer’s instructions. The Abbott RealTime HCV Genotype II is an in vitro reverse transcription-polymerase chain reaction (RT-PCR) assay for the qualitative identification of HCV genotypes 1, 1a, 1b, and 2–5 in plasma or serum from individuals chronically infected with HCV. Tumor necrosis factor α (TNF-α) and interleukin 6 (IL-6) were measured using the automated ELISA technique (Protein Simple 3001 Orchard Parkway San Jose) [40].

### 2.5. Liver Elastometry

Each patient underwent an ultrasound scan before starting antiviral treatment and within one month of achieving viral eradication. Elastometry with ARFI measurements were obtained using a Siemens Acuson S2000 ultrasound system (Siemens Medical Solutions, Issaquah, WA, USA) by the same experienced sonographer (ADS). Patients were placed in the supine position and underwent ARFI on B-mode imaging during expiration, after fasting overnight. A region of interest in the liver parenchyma, free of large blood vessels, was selected using the intercostal approach. Ten consecutive successful measurements were performed in each patient and the mean value was used to calculate the stiffness expressed in kilopascals using Young’s modulus (kPa = 3Vs2).

### 2.6. Statistics

The Statistical Package for Social Sciences, SPSS 27.0, was used (SPSS, Inc., Chicago, IL, USA). Variables were tested for normality of distribution using the Shapiro–Wilk test. Data are expressed as mean values ± SD (normally distributed variables) and as median values and interquartile ranges (non-normally distributed variables). Variables were log-transformed when non-normally distributed. Pearson’s correlation coefficient was used to investigate the level of association between continuous variables. For paired samples, Student’s *t*-test and Mann–Whitney U-test were used to assess differences between baseline and post-treatment.

Based on FGF-21 levels of patients with liver disease and the observed reduction following DAAs reported in the literature, assuming a power of 0.80 and alpha of 0.05, 37 patients were considered appropriate to highlight an expected clinically relevant FGF-21 change of 35%. The number of subjects was further increased to 45 foreseeing a dropout rate of 20%.

## 3. Results

Our study enrolled and followed up with 45 patients, effectively achieving a 100% follow-up rate. The cohort was predominantly male (80%), with a mean age of 63 ± 12 years, highlighting the demographic most affected by HCV-related liver disease. Additionally, 28.9% of the patients had cirrhosis, and 8.9% had diabetes, underlying the complexity and comorbidity burden in our study population. A breakdown of baseline characteristics is extensively detailed in Table 1.

At baseline, we observed noteworthy correlations between liver stiffness and certain biochemical markers. A strong negative correlation was seen with albumin levels (R = −0.642, *p* = 0.007), confirming that lower albumin is an indicator of more advanced liver disease. Similarly, liver stiffness negatively correlated with platelet count (R = 0.598, *p* < 0.001), echoing the existing literature that lower platelet counts can signal liver damage.

Conversely, liver stiffness positively correlated with aspartate aminotransferase (AST; R = 0.566, *p* = 0.001) and alanine transaminase (ALT; R = 0.434, *p* = 0.012), both being markers typically elevated in hepatic dysfunction. However, liver stiffness did not demonstrate a statistically significant correlation with other variables such as HCV-RNA levels (*p* = 0.623), disease duration (*p* = 0.464), FGF-21 (*p* = 0.432), BMI (*p* = 0.252), or age (*p* = 0.695).

**Figure 1:**
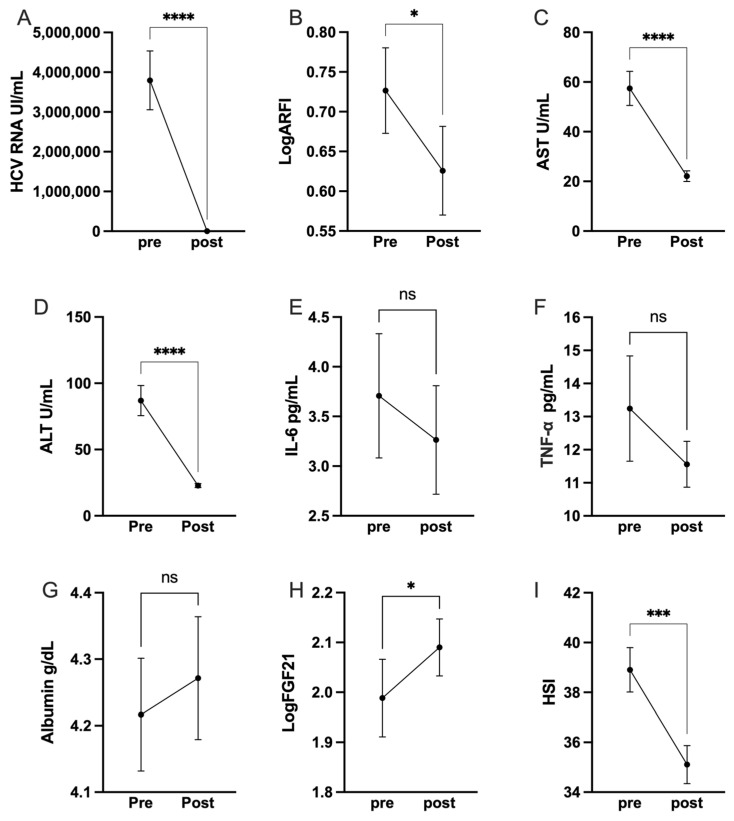
HCV-RNA (**A**), liver stiffness (**B**), and AST (**C**) and ALT (**D**) are significantly reduced following viral eradication. IL-6 (**E**), TNF alpha (**F**), and albumin (**G**) were not significantly changed, while FGF-21 (**H**) exhibited an increase, and hepatic steatosis index a decrease (**I**). ns, not significant; * *p* < 0.05, *** *p* < 0.001, **** *p* < 0.0001.

Post-treatment with DAAs led to substantial clinical improvements. The complete negativization of HCV-RNA levels in all patients after DAA treatment is a striking endorsement of antiviral efficacy (Figure 1A). Correspondingly, there was a marked reduction in liver stiffness from 7.1 ± 6.6 kPa to 4.9 ± 2.7 kPa (*p* = 0.027; Figure 1B). AST and ALT, which are traditionally used as markers of liver health, showed significant improvements (*p* < 0.0001; Figure 1C,D). This overall profile illustrates the dual benefit of viral clearance and hepatic recovery attributable to DAA therapy.

However, it is crucial to acknowledge the lack of significant reduction in pro-inflammatory cytokines like IL-6 and TNF-α (*p* = 0.52 and *p* = 0.23, respectively; Figure 1E,F), and the non-significant increase in albumin levels (Figure 1G). This opens up an avenue for future studies to investigate other therapies that could work in tandem with DAAs to lower these inflammatory markers. The increase in FGF-21 levels post-treatment (*p* = 0.019, Figure 1H) adds an additional layer of complexity to our understanding of liver pathology and requires further exploration.

The analysis of treatment-induced changes revealed a significant negative correlation between the liver stiffness reduction and FGF-21 (R = −0.616, *p* = 0.033) and albumin changes (R = −0.725, *p* = 0.003). This suggests that increases in FGF-21 and albumin levels may be beneficial indicators for assessing the treatment response concerning liver stiffness. We also observed a trending but non-significant correlation with AST (R = −0.433, *p* = 0.1), suggesting a trend that might become significant with a larger sample size. Moreover, no correlation was identified between the liver stiffness reduction and changes in ALT, INR, or platelet count.

Our study reported a remarkable improvement in hepatic steatosis, as indicated by the hepatic steatosis index (HSI). The HSI score dropped significantly from 39.31 to 35.25 (*p* < 0.001; Figure 1I), marking a promising trend in the amelioration of fatty liver conditions. However, it is important to note that no significant correlation was found between the improvement in hepatic steatosis and FGF-21 changes (*p* = 0.87), suggesting these may be independent events in the context of HCV eradication.

## 4. Discussion

We herein report that patients with HCV-related liver disease undergoing eradication with DAAs experience, as expected, a reduction in liver stiffness coupled with improvement in well-established markers of liver disease. We report an increase in FGF-21 serum levels upon HCV eradication, expanding on data from a previous in vitro study reporting a negative independent correlation between hepatic FGF-21 mRNA and hepatic HCV-RNA levels in livers from patients with HCV [13]. Interestingly, and as largely documented in the literature, the authors also found that the FGF-21 transcriptional regulator PPAR-α’s expression was also decreased in HCV-infected patients compared with normal controls, further supporting our finding [13,15,16]. Conversely, our report contrasts with the only other available clinical study analyzing serum FGF-21 in response to HCV eradication: in 2018, El Sagheer et al. reported higher FGF-21 levels in patients with genotype 4 chronic hepatitis C infection and described a reduction after viral eradication [31]. These two contrasting outcomes may be explained by the different times at which FGF-21 was assessed and different definitions of SVR. Unlike our study, El Sagheer et al. described SVR as undetectable HCV-RNA 12 weeks after termination of treatment. It could be reasonable to think that FGF-21 may experience a reduction in the early post-treatment phases thanks to the termination of the cytotoxic stimulus, followed by a gradual increase as liver function is gradually recovered. A high concordance between SVR12 and SVR24 has been reported, as the majority of virologic relapses occur within 12 weeks after the end of treatment [41]; however, this may not reflect the time needed by the liver to regain its complete functionality. Although these data provide interesting insight into the trend of FGF-21 in the setting of a chronic illness and may link FGF-21 to the synthetic capability of the liver, they do not clarify the precise stimuli inducing FGF-21.

Another possible explanation as to why FGF-21 levels increase after HCV eradication may lie in the concept of FGF-21 resistance, a condition already described, though not free of controversy, in murine models of obesity [42,43]. It appears that in obese mice, FGF-21 is persistently elevated while FGF-21 receptor complexes are underexpressed, and the signaling pathway is blunted [44]. It could be reasonable to think that, unlike acute insults, such as acute or fulminant hepatitis, where FGF-21 peaks at high levels [29], in the setting of a chronic infection, FGF-21 manifests an initial peak followed by a persistent, moderate increase as a state of FGF-21 resistance progressively develops [45,46]. To confirm this hypothesis, the expression pattern of FGF-21 receptors should be evaluated throughout the course of the infection as well as at different times during treatment and after its completion. The elevation of FGF-21 at SVR24 may represent a resolution of the state of FGF-21 resistance and an attempt of the liver to fully repair itself.

An additional novel finding of this study was that changes in liver steatosis, as reflected by the surrogate marker HSI, were not correlated with FGF-21 changes, despite previous studies suggesting that FGF-21 reflects liver fat accumulation. The first study to describe FGF-21 levels in CHC patients, dating back to 2012, reported higher values in those infected with respect to healthy subjects. Within those suffering from chronic infection, the levels were highest in those presenting with steatosis and were correlated with steatosis grade [47]. Even in the absence of chronic hepatitis infections, FGF-21 has been described as a metabolic marker, correlating with the degree of steatosis [48]. We hypothesize that the lack of correlation between FGF-21 and steatosis may be due to the presence of a hierarchy of insults influencing FGF-21 levels. In the setting of moderate to advanced fibrosis, FGF-21 may lose its power as a predictive marker of steatosis as it would mostly be affected by ECM deposition. An additional consideration in regards to the lack of correlation between HSI and FGF-21 is that we did not try to differentiate between patients with viral steatosis due to the cytopathic effect of the virus, which are those that would exhibit the most significant reduction in steatosis in response to treatment, and those with metabolic steatosis due to a concomitant metabolic syndrome, which would only exhibit a modest decrease in fatty liver accumulation following viral eradication. Although overall we did witness a reduction in steatosis following HCV eradication, its lack of correlation with FGF-21 may be due to confounding factors related to a divergence in the pathogenetic mechanisms, which, despite sharing parts of the molecular pathway, present different clinical and prognostic implications. This, however, may be in favor of a hierarchical theory of insults so that, as the infection clears out, FGF-21 reflects an improvement in viral steatosis firstly, and only afterwards an improvement in metabolic steatosis. Finally, the effect of DAAs on steatosis is still unclear, as some data report its progression despite HCV eradication, especially in metabolically deranged patients and in PNPLA3 variants [49,50]. It may be in this very subset of patients that drugs targeting specific players in molecular pathways involved in lipid metabolism, such as the PPAR-α–FGF-21 axis, could be of benefit. If this was confirmed, then FGF-21 agonists could be potentially introduced as an add-on to DAAs in specific subsets of patients, in whom the metabolic risk associated with liver steatosis remains elevated regardless of viral eradication.

Similar to albumin, FGF-21 was found to be correlated with improvement in liver stiffness, suggesting a potential role of FGF-21 as a non-invasive marker of stiffness. However, a different study reported no association between the levels of FGF-21 and fibrosis stage in chronic hepatitis B infection [51]. Therefore, the utility of FGF-21 as a potential marker of fibrosis and the effect of different etiologies and of different levels of fibrosis on FGF-21 should be further studied. Additionally, the potential role of FGF-21 in fibrogenesis and its resolution requires better characterization. Indeed, FGF-21 administration in animal models has resulted in an improvement of fibrotic status, via multiple mechanisms, and in a decrease in pro-inflammatory cytokines such as IL-6 and TNF-α [52]. However, this study failed to identify any correlation between FGF-21 and pro-inflammatory markers, before and after treatment eradication.

As expected, we report a decrease in liver transaminases upon viral eradication; however, no correlation was found with the improvement in liver stiffness. A possible explanation is that, although included in a number of non-invasive algorithms for the detection of fibrosis, AST and ALT alone lack the sensitivity and specificity to accurately detect and estimate liver fibrosis [53]. Transaminases are elevated when disease processes affect liver cell integrity and are direct markers of hepatic cytotoxicity; on the other hand, in the setting of fibrosis, they are indirect markers and reflect alterations in hepatic function, or rather dysfunction.

Yang et al. proposed FGF-21 as a marker of the functional status of mature/differentiated or phenotypically normal hepatocytes during the process of liver injury, recovery, and pathogenesis. In their study, the expression of FGF-21 was significantly induced in the liver following reversible perturbation, such as partial hepatectomy, regeneration, hepatic steatosis, as well as irreversible hepatic damage from chronic hepatitis, cirrhosis, and chemical hepatocarcinogenesis in both animal models and human patient samples. They suggested that FGF-21 is expressed in association with the loss of the normal functional capacity of hepatocytes due to pathogenic processes [28]. Additionally, a study conducted on mice described FGF-21 as being induced following acetaminophen-related acute hepatotoxicity; furthermore, circulating FGF-21 levels significantly increased as early as 3 h, before AST and ALT [54]. Should our findings be confirmed by further studies, FGF-21, unlike albumin, may be a potential marker of both fibrosis and hepatic cytotoxicity.

Finally, FGF-21 is elevated in illnesses characterized by mitochondrial damage [55], which is a prominent feature of HCV infection. In the setting of chronic infection, ultrastructural changes, alterations in electron transport, and excess reactive oxygen species production occur. These mitochondrial abnormalities correlate with disease severity and resolve with viral eradication [56]. The initial FGF-21 decrease, in the early post-treatment phases, may reflect the reversibility of mitochondrial damage upon the removal of noxious stimuli, whereas the following, later-onset increase in FGF-21 may reflect a complete return to hepatic health and functionality.

Our study presents some limitations. First of all, FGF-21 was evaluated only at T0 and at T1, 9 or 12 months after the eradication of chronic HCV infection; no other assessment was made at a later time. Additional measurements would be required to properly characterize the trend of FGF-21 and how it correlates with full hepatic histological recovery. However, our evaluation was the latest ever conducted in this setting, shedding light on the long-term trend upon sustained viral eradication. Additionally, HSI, although used in this study as a non-invasive marker of steatosis, lacks validation in the setting of HCV infection, as it has only been extensively studied in NAFLD [39]. However, no indirect marker of steatosis is currently validated in such a setting. Finally, our sample population, although well representative in terms of age, was disproportionately composed of male subjects. Nonetheless, according to the latest epidemiological data, this imbalance is reflective of the current gender distribution in Europe.

Our study also presents some strengths. Unlike previous studies, several genotypes were included in this study, as well as variable degrees of liver impairment, from mild fibrosis to cirrhosis. We decided to include different levels of liver impairment and fibrosis to evaluate whether FGF-21 levels could be affected by a decreased synthetic ability of the liver seen in more advanced stages of the disease.

## 5. Conclusions

In conclusion, we report that serum levels of FGF-21 increase 24 weeks after DAA-mediated viral eradication. We hypothesize that this result, contrasting with previous clinical data but agreeing with previous in vitro models, might be attributed to the different times at which FGF-21 was assessed post-treatment. The initial decrease may be indicative of a reduction in hepatic damage due to viral eradication, whereas the subsequent increase may be suggestive of the gradual recovery and improvement in the synthetic ability of the liver, as well as the lack of suppression of the PPAR-α pathway induced by the presence of the virus. Further studies are needed to shed light on the several aspects still to be elucidated. The evaluation of FGF-21 in different disease states could add important information. Similarly, evaluation in a state of severe, advanced fibrosis would be needed; as one surpasses a certain threshold of liver dysfunction, it would be reasonable to think that FGF-21 production may be impaired. Finally, confirmation and further clarification on the pathogenetic mechanisms inducing FGF-21, via an integration of biochemical, histological, and clinical investigations, would be required in order to understand whether FGF-21 passively changes or is an active player in the pathogenesis of fibrosis. This could provide relevant data on how to exploit FGF-21 as a potential marker of liver disease and as a therapeutic strategy.

## Figures and Tables

**Table 1:** Characteristics of study population before and after treatment with DAA.

	Mean ± SD, Median (IQR), % or N (*n* = 45) before DAA	Mean ± SD, Median (IQR), % or N (*n* = 45) after DAA	*p*
Age (years)	63 ± 12	63 ± 12	
BMI (kg/m^2^)	26.2 ± 3.5	26.5± 3.5	
Disease duration (years)	18 ± 10	18 ± 10	
HCV-RNA (UI/mL)	3,794,855 ± 4,849,106	Undetectable	<0.0001
ARFI (kPa) *	7.12 (6.6)	4.9 (2.7)	0.027
FGF-21 (ng/mL) *	156 (118)	168 (128)	0.019
ALT (U/L)	86.9 ± 74.9	22.7 ±9.1	<0.0001
AST (U/L)	57.4 ± 45.0	22.1 ± 13.4	<0.0001
Albumin (g/dL)	4.22 ± 0.38	4.26 ± 0.43	0.47
TNF-α (pg/mL)	13.2 ± 6.74	11.56 ± 2.93	0.23
IL-6 (pg/mL)	3.8 ± 2.8	3.34 ± 2.48	0.52
HSI	39.31 ±5.01	35.25 ± 4.25	0.0004
Male (%)	80	80	
Cirrhosis (%)	28.9%	28.9%	
Diabetes (%)	8.9	8.9	
Unspecified HCV Genotype (N)	4	4	
HCV Genotype 1 (N)	20	20	
HCV Genotype 2 (N)	16	16	
HCV Genotype 3 (N)	2	2	
HCV Genotype 4 (N)	2	2	
HCV Genotype 5 (N)	1	1	

SD, standard deviation; IQR, interquartile range; N, number; BMI, body mass index; ARFI, acoustic radiation force impulse; FGF-21, fibroblast growth factor 21; ALT, alanine transaminase; AST, aspartate aminotransferase; TNF-α, tumor necrosis factor α; IL-6, interleukin 6; HSI, hepatic steatosis index. * non-normally distributed; *p* is from a paired *t*-test.

## Data Availability

Data will be made available upon reasonable request to the corresponding author.
